# *Pneumocystis jirovecii* Transmission from Immunocompetent Carriers to Infant

**DOI:** 10.3201/eid1407.071431

**Published:** 2008-07

**Authors:** Laura Rivero, Carmen de la Horra, Marco A. Montes-Cano, Alfonso Rodríguez-Herrera, Nieves Respaldiza, Vicente Friaza, Rubén Morilla, Sonia Gutiérrez, José M. Varela, Francisco J. Medrano, Enrique J. Calderón

**Affiliations:** * Virgen del Rocío University Hospital, Seville, Spain; † Network Center for Biomedical Research in Epidemiology and Public Health, Seville, Spain

**Keywords:** Pneumocystis, epidemiology, transmission, dispatch

## Abstract

We report a case of *Pneumocystis jirovecii* transmission from colonized grandparents to their infant granddaughter. Genotyping of *P. jirovecii* showed the same genotypes in samples from the infant and her grandparents. These findings support *P*. *jirovecii* transmission from immunocompetent carrier adults to a susceptible child.

*Pneumocystis jirovecii* is an atypical fungus that causes pneumonia in immunosuppressed persons; many questions about its epidemiology and transmission remain unanswered (*1*,*2*). Animal sources for *P. jirovecii* can be excluded because the *Pneumocystis* organisms that infect mammalian species are characterized by strong, close host-species specificity (*3*). Similarly, an environmental reservoir of infection has not been found (*4*). Airborne transmission has been demonstrated in animal models, but the route of transmission of *Pneumocystis* organisms among humans is unclear (*5*). *P. jirovecii* DNA has been identified in ambient air, and airborne transmission between humans is likely (*4*). This hypothesis is supported by reports of case clusters of pneumocystis pneumonia (PcP) among immunosuppressed patients, transmission of *Pneumocystis* DNA from PcP patients to healthcare workers, and transmission of *Pneumocystis* infection from a mother with PcP to her susceptible child (*6*–*10*).

Use of highly sensitive PCR technologies has enabled detection of low levels of *P. jirovecii* in respiratory samples from persons who do not have PcP. Many terms—colonization, carriage, asymptomatic infection, and subclinical infection—have been used to describe these findings. Studies have shown that persons who have underlying HIV infection or other causes of immunosuppression and those who are not immunosuppressed but have chronic lung disease may often be colonized by *P. jirovecii* (*11*–*13*). Further hypotheses claim that these groups may play a role in person-to-person transmission and that they may serve as reservoirs for future *Pneumocystis* infection in other susceptible persons; however, this hypothesis has not been proven.

## The Study

A 6-month-old female infant was admitted to Virgen del Rocío University Hospital, Seville, Spain, with a history of nonproductive cough and difficulty breathing. She had been born by vaginal delivery after 40 weeks of gestation, birth weight was 3,490 g, and she had been breast-fed for 2 months. Her mother was healthy and HIV negative. At the time of examination, the infant was afebrile, weighed 4.5 kg (<3rd percentile), and was 62 cm long (<3rd percentile). Respiratory rate was 70 breaths/min; oxygen saturation (by pulse oximetry) was 89%. Fine crackles were heard in both lungs. She had neither lymphadenopathy nor visceromegaly. Diagnostic testing found leukocyte count 12,600 cells/mm^3^, CD4+ cells within normal limits, and no immunosupression. Serologic and molecular test results for HIV infection were negative. Chest radiograph showed diffuse interstitial infiltrates suggestive of PcP.

*P. jirovecii* DNA was detected in nasopharyngeal aspirate samples by amplifying the mitochondrial large-subunit gene of rRNA with nested PCR. No other infections were detected by culture, molecular tests, or serologic tests. The infant was treated with high-dose trimethoprim-sulfamethoxazole and adjuvant steroids. She did well and was discharged a month later.

To determine the origin of the infant’s infection, we investigated all persons who lived with her, i.e., parents, brother, and grandparents. Each person underwent clinical and epidemiologic examination and submitted oropharyngeal samples for analysis. Informed consent was obtained from all persons, and the study was approved by the hospital’s ethics committee.

The infant’s mother, father, and brother were healthy. Her grandmother and grandfather reported a history of rheumatoid arthritis and chronic bronchitis, respectively. None had pneumonia symptoms at the time of the study.

Identification of *P. jirovecii* colonization was carried out by analyzing oropharyngeal samples with nested PCR at the gene encoding the mitochondrial large-subunit rRNA, with primers pAZ102-E and pAZ102-H in the first-round amplification, followed by pAZ102-X and pAZ102-Y in the second-round amplification (*12*). *Pneumocystis* DNA was extracted after samples were digested with proteinase K at 56ºC by using a commercial kit (QIAGEN, Hilden, Germany). To prevent contamination, pipettes with filters were used for all manipulations. DNA extraction and preparation of the reaction mixture were performed in 2 different rooms under separate laminar-flow hoods. PCR and analysis of PCR products were performed in another room. Controls were run simultaneously with respiratory samples. Positive controls were bronchoalveolar lavage specimens from PcP patients; negative controls were autoclaved water in the PCR mixture in the absence of the DNA template controls.

All samples that were positive according to nested PCR were sequenced; polymorphisms at nucleotide positions 85 and 248 were detected by direct sequencing (*12*). The nested PCR products were purified by using Sephacryl S-400 columns (Amersham Pharmacia Biotech AB, Uppsala, Sweden) and reamplified with ABI Prism dRhodamine Terminator Cycle Sequencing Ready Reaction Kit (PE Applied Biosystems, Foster City, CA, USA). Then for each reaction, 5 μL of PCR product, 4 μL of terminator ready reaction mix, and 3 pmol/L of primer were added. The extension products were purified by ethanol precipitation procedure to remove the excess dye terminators. Each sample pellet was resuspended in 12.5 μL of template suppression reagent and heated at 95ºC for 3 min to denature the product. Electrophoresis was carried out on the ABI prism 310 sequencer (PE Applied Biosystems) in accordance with the manufacturer’s recommendations. The sequenced DNA fragments were analyzed by Sequence Navigator version 1.0.1 (PE Applied Biosystems).

*P. jirovecii* DNA was found in oropharyngeal samples from the infant’s grandparents but not her parents or brother. Genotype 1 (85C/248C) was identified in the infant and in her grandparents. Moreover, coinfection with genotype 3 (85T/248C) was detected in the grandfather. In addition, *P. jirovecii* dihydropteroate synthase locus was analyzed in the samples from the infant and her grandparents by PCR restriction fragment-length polymorphism, as described (*12*). Wild dihydropteroate synthase genotype was detected in all samples.

## Conclusions

This study provides molecular evidence of *P. jirovecii* transmission from human immunocompetent asymptomatic carriers to a susceptible host, who developed PcP. We cannot exclude the possibility that the cases described were infected by the same environmental source; however, an exosaprophytic form of *P. jirovecii* has not been found (*4*).

*P. jirovecii* colonization has been shown in pregnant women, and their role as contagious sources for their susceptible newborn infants has been suggested (*14*). In our case, mother-to-infant transmission can be ruled out because the infant’s mother was not colonized by *P. jirovecii*. An alternative explanation, but less probable considering the time course of the clinical symptoms, is that the infant acquired the infection in the hospital during delivery and was the source of infection for her grandparents. However, her grandfather was colonized by genotypes 1 and 3, and the infant had only genotype 1.

We hypothesize that the infant was infected by *P.* *jirovecii* through close contact with her grandparents because they looked after the child full time and lived in the same house. In comparison with animal model experiments on transmission of *Pneumocystis* infection (*5*), the airborne transmission of *P. jirovecii* from the grandfather to the grandmother and the infant is the most probable explanation, especially in view of the high prevalence of *P.* *jirovecii* colonization of persons with chronic bronchial disease in our area and the grandfather’s sputum production associated with this condition (*15*).

This study provides molecular evidence that transmission of *P. jirovecii* from colonized immunocompetent carrier hosts to susceptible persons may occur. The role of persons with chronic pulmonary disease who are colonized with *P. jirovecii* as major reservoirs and sources of infection warrants further investigation.
